# Medical science, culture, and truth

**DOI:** 10.1186/1747-5341-1-13

**Published:** 2006-12-19

**Authors:** Grant Gillett

**Affiliations:** 1 Bioethics Centre, University of Otago, Dunedin, New Zealand

## Abstract

There is a fairly closed circle between culture, language, meaning, and truth such that the world of a given culture is a world understood in terms of the meanings produced in that culture. Medicine is, in fact, a subculture of a powerful type and has its own language and understanding of the range of illnesses that affect human beings. So how does medicine get at the truth of people and their ills in such a way as to escape its own limited constructions? There is a way out of the closed circle implicit in the idea of a praxis and the engagement with reality that is central to it and the further possibility introduced by Jacques Lacan that signification is never comprehensive in relation to the subject's encounter with the real. I will explore both of these so as to develop a conception of truth that is apt for the knowledge that arises in the clinic.

## Background

"Medicine has for long possessed the qualities necessary to make a science. These are a starting point and a known method according to which many valuable discoveries have been made over a long period of time [1]."

The known method of the Hippocratics was rooted in healing practice, spurred by the actuality of human suffering, and it built on folk medicine by careful documentation of cases, interventions, and the effects of those interventions. Such a method cautiously accumulates a conceptual and theoretical apparatus influenced by the culture in which it has grown. In fact it gives rise to its own "medical culture" and a discursive regime that is in a position to become privileged as medicine achieves institutional status and political power. The result can be a quite restrictive view of what counts as truth such that the science becomes increasingly normalised as a cultural production. The regime of truth that develops defines its own scientific standards, disciplines, rules, and methods of validation and can effectively exclude all apart from the orthodox and privileged.

Truth therefore needs to reserve for itself the possibility of subversion or escape from the closed discursive circle that potentially restrict s the gaze and the imagination of the seekers after truth, it needs, in a word, a dynamic encounter with reality of the type we can make sense of after the philosophical semantics of Jacques Lacan [1]. I will argue that if we broaden the notion of *trauma *from its psychoanalytic origins we can understand the "traumatizing" of the closed world of language and truth by the incursion of the medical encounter as it occurs in the presences and silences of the clinic. This, I will claim gives rise to a form of *repetition *that is the midwife of the will to truth (or more adequate untruth). But this journey can only begin if we recognise the potential impasse faced by any scientific endeavour.

## Truth, science, and culture

Medical science has followed the ideal of scientific objectivity so that we make observations, offer theories about the realities they indicate and assess those theories in the light of our ongoing accumulation of clinical and scientific evidence. But, as Kuhn and others have argued, the paradigm within which we work licenses certain evaluative judgments about the evidence and the means by which it is obtained which are themselves nested within its own structure of belief such that the formation of any scientist requires one to be inducted into "a strenuous and devoted attempt to force nature into the conceptual boxes supplied" [2]. In medicine (as in its parent approach to scientific objectivity) we cling to the dogma that evidence is by nature true whereas theory is inference and therefore subject to flaws in reasoning even though it may, by successive refinement, approximate truth. But Feyerabend raises a voice of dissent;

Experience arises together with theoretical assumptions not before them, and an experience without theory is just as incomprehensible as is (allegedly) a theory without experience: eliminate part of the theoretical knowledge of a sensing subject and you have a person who is completely disorientated and incapable of carrying out the simplest action. Eliminate further knowledge and his sensory world (his 'observation language') will start disintegrating. [3]

These points are familiar to post-Kantian philosophers but run counter to a belief in scientific realism according to which the world is the way science says it is because there is a transparent and simply verifiable correspondence between our scientific descriptions and the reality they describe. On this view, science reveals the nature of the world and the processes going on within it and discloses facts about human health and disease. It comes as a surprise to many medical scientists that there are significant shifts in scientific theory: "viewed *sub specie eternitatis *scientists (even physical scientists) are a fickle lot. The history of science is a tale of multifarious shiftings of allegiance from theory to theory" [4].

Such arguments in contemporary philosophy of science, critique the idea that medicine is an interconnected "totality of truths equally open to all scientific inquirers who may share their techniques and experiences" [5]. The truths concerned begin to look more like a cultural product of "normal medical science" than we often admit.

Hacking identifies seven "styles of scientific reasoning" that illustrate both the diversity and interconnectedness of the web of scientific belief: (a) *postulates and axioms *such as the idea that discernible structure underpins and explains function. Structure is investigated by increasingly sophisticated methods such as electron microscopy and nuclear magnetic resonance scanning but we do not question the thought that we can and do identify that which gives rise to the diversity of medical phenomena. We conjecture that the macroscopic and microscopic structures reveal the causes of human function and dysfunction rather than any top-down or holistic determination of structure by context (for instance in embryology or psycho-neuro-immunology [6].

*Experimental exploration and measurement *are interwoven with the investigation of structure and function and the thought that experimental data constitute the objective, self-declaring foundations of biomedicine. But Feyerabend warns us that this data and its objectivity are coeval with the theoretical structures motivating its capture.

*Hypothetical modelling *is related to and in "reflective equilibrium" with other attitudes and techniques defining medicine. It shades into axiomatic thinking to generate models such as "immune surveillance", neoplastic transformation, the genesis of atherosclerosis, and the axes of psychiatric diagnosis. In each case (for instance in psychiatric diagnosis) an overarching thesis (such as the idea that a disorder is an internal derangement of an individual and understandable in terms of individual departure from biological norms) conditions the kinds of data that are sought and looked for to explain medical phenomena.

*Medical taxonomy *is founded on the idea of natural kinds [7,8], whereby general terms are taken to designate phenomena naturally occurring in the actual world, such as gold, tigers, water, oxidation, and white blood cells. These terms "carve nature at its joints" underpinning a realist metaphysics. But we should notice that taxonomic categories reflect the best current scientific knowledge in the relevant area so that the linguistic community defers to the scientific experts and therefore the category becomes *de facto *a product of orthodox scientific theory. Therefore a radical and serious challenge to the scientific theory applied to the phenomena in question has metaphysical implications and can affect even basic questions about when a certain scientific kind or phenomenon (such as Oxygen) was discovered and by whom [9].

There is a vigorous debate about whether diseases are natural kinds (in the sense defined) and represent objectively specifiable, dysfunctions of the organism they affect [10] because some would argue that classifying a bodily condition as a disease involves both describing and evaluating the state of an organism [11-13]. If that is so, our claim to scientific objectivity has to concede that the terms in which we do the science are infused with evaluations and potential sources of selective bias.

*Statistical analysis *therefore appears in a different light because, as a method of finding non-coincidental correlations between phenomena – thereby linking taxonomic categories – it is subject to the uncertainties and qualifications applicable to the categories. These statistical associations may be supported by, for instance, showing that drugs with known effects on certain biochemical processes have a statistically demonstrable influence on the course of disease. Indeed that conjunction of methods is often used to derive a patho-physiological story about disease, but notice how dependent it is on what we choose to measure, count as significant, and have interventions for.

*Historical or genetic explanation *of the occurrence of a pathological abnormality in terms of bodily structure and function is our way of understanding diseases and their therapy. The relevant explanations are tied to our conceptions of natural kinds, and laws of nature, and therefore, again, to the models and taxonomies central to the web of belief that is contemporary biomedicine.

*Laboratory science *is a growing part of medical science as the means of isolating and investigating the processes and events that lie behind (clinical) phenomena and it gives operational effect (and thereby technological or mathematical legitimacy) to our scientific taxonomies.

This mix of theory and methodology yields a preferred description for (and means of documenting or measuring) every disease, composed from resources provided by the paradigm (or network of theory, technology, and practices) of contemporary clinical biomedicine. The enterprise is Hippocratic in that it is pragmatic, causal and naturalistic, based on observation, and grounded in a scientific model of human function but it is also transformed and encultured by reductive biomedicine emerging from enlightenment aspirations to exhaustive and complete Truth about the world and everything in it including human health and disease.

Consider an important example – subarachnoid haemorrhage (SAH) due to a berry aneurysm on one of the cerebral arteries (a berry aneurysm is an out-pouching of a cerebral artery caused by a deficiency in the arterial wall). We believe that increasing blood pressure during life causes the aneurysm to stretch and then split. The aneurysm ruptures causing a haemorrhage around and sometimes into the brain which can be fatal. We detect the haemorrhage and the aneurysm by imaging the brain and its blood vessels. Then we try and obliterate the aneurysm to prevent recurrent haemorrhages.

This problem and its resolution is a testimony to the practice of meticulous post-mortem examination of people who have died from strokes, the development of imaging techniques to reveal structures inside the head, theoretical modelling of a pathological process, and the development of neurosurgical techniques (and supporting therapies such as analgesia, antisepsis, anaesthetics, and pharmacology). Despite its technical complexity this is a comparatively simple conceptual exercise aimed at an anatomical anomaly and the problems arising when one tries to correct it. But aspects of the problem elude us. Why does an aneurysm split and bleed when it does? What causes the vaso-spasm that sometimes follows SAH and causes a disabling stroke? Why do some people recover well and others badly? These questions, like many arising in the clinic from the inadequacy of our responses to human suffering, are not well addressed by contemporary bio-medical science, and yet the scientific approach of positivist biomedicine remains dominant both in treatment and in funded research into the conditions presenting themselves for medical attention. We do fund a search for the chemicals in the cerebro-spinal fluid that might promote vaso-spasm. We do fund neuro-cognitive tests of memory, executive function, speech, and so on. We do look at drugs thought to mitigate this or that source of damage in such an event. All these technologies focus on a biomedical construction of SAH and there is a powerful coalition between medical science and the researched medicines industry in pursuing them. What of other healing methods – meditation, natural foods, herbal treatment, or the disciplines aimed at the health of the soul? These have no place in our conceptions of SAH, so they get a mention neither in our science nor in our clinical methodology. We are not open-minded about SAH as a human phenomenon, we conceive it on the model of the human machine and its dysfunctions and if the truth about susceptibility to SAH (or heart disease, or diabetes, or schizophrenia) lies beyond such conceptions, we are blind to it.

## Medical culture and its discursive regime

To understand why such an open position is hard to attain we need to examine the culture that is biomedicine. We can begin with Foucault's account of the relationships between social, political, and economic power and Truth (the capitalization is important when discussing Scientific realism).

In societies like ours, the "political economy" of truth is characterized by five important traits. "Truth" is centered on the form of scientific discourse and the institutions which produce it; it is subject to constant economic and political incitement (the demand for truth, as much for economic production as for political power): it is the object, under diverse forms, of immense diffusion and consumption (circulating through apparatuses of education and information whose extent is relatively broad in the social body, notwithstanding certain strict limitations); it is produced and transmitted under the control, dominant if not exclusive, of a few great political and economic apparatuses (university, army, writing, media); lastly, it is the issue of a whole political debate and social confrontation ("ideological" struggles). [14]

These features define the culture of medical science as it arises from and within medical institutions, knowledge, and political alliances.

### Medicine clearly locates its truth within scientific discourse

The entire move towards Evidence Based Medicine (EBM), laudable as it is, has recast medical knowledge such that experimental studies designed to isolate single effective interventions (perhaps acting in combination) take on the highest status and, by so doing, lock in place a reductive model of health and disease.

### It is incited to further efforts to expand and use that truth from within and without

The focus on EBM means that the knowledge of health and disease is constructed in terms of what might lead to effective interventions that can be manufactured, marketed, and applied to the human body. This focus influences clinical explanation and therefore the ends toward which diagnosis is oriented.

### It is diffused and consumed in a huge number of different ways

Medical thinking (and low level medically influenced advice and "pop wisdom") are everywhere in Western society – magazines, television, films, and advertising. None of these sources undertake intelligent deconstruction or critique – they evince the "sound-bite" mentality whereby any map of the health care terrain is "dumbed down" into simplistic terms – condition X is treated by drug Y (from company Z).

### The practice of medicine is dominated by methodologies sponsored by the big players

The alliance between major pharmaceutical concerns, the institutions performing medical research, government regulatory bodies setting standards for health care, and the organized profession instances Foucault's "political and economic apparatus" and reinforces the accepted scientific view of health and disease (which has, to be fair, led to nearly all the major advances in health care, particularly in the Twentieth Century). These alliances identify good quality care with the latest and most expensive medical technology and therefore undermine clinical acumen (a particular clinical synthesis by an experienced physician) in favour of objective indicators based on the general features of the case and a recipe of therapy. This conception of good clinical practice *makes *physicians use the latest technology because reliance on their own, perhaps very experienced, judgment could leave them exposed to medico-legal risk if, as is bound to happen at least sometimes, things do not turn out well.

### It is a major focus of political and social debate

Modern medicine is part of a discursive regime in which those who live and die within it are measured and of immense social importance. Clinical medicine, arm in arm with science and the political weight of the profession, dominates the debate about health care provision by any society, a debate with symbolic significance for any caring society, political relevance to the controllers of society, and huge economic implications.

The cultural context outlined is seen par excellence in the conceptual structure of the DSM system of psychiatric classification. DSM represents a taxonomy of mental illness primarily conceived as a range of disorders of the individual which can be diagnosed on the basis of an objective checklist of features and thereby fitted, as best the mental disorder of any individual can be, into a general classification susceptible to documentable interventions. In the broader context of the critical claim that the range of maladies of the soul should be as detailed as the range of souls who suffer [15], the DSM seems to perpetuate a cultural orientation towards meaning-giving in the context of mental distress that is conditioned by aspirations to self-constitute psychiatry on the model of disease locked into the natural sciences (rather than the mental or moral sciences).

It is this culture, and biomedical science as one of its productions, that is vulnerable to the critiques I have mentioned. Kuhn's claim that science is not merely the gradual accumulation of truth to provide a unified structure of knowledge which encompasses natural phenomena but rather is marked by revolutions in which old paradigms are attacked, ravaged, and discarded or abandoned through creeping disaffection so that new paradigms take their place. He noted that the reasons for paradigm shift may not reflect debates within "the logical structure of scientificknowledge" [16] itself, of course, a cultural production. He argued that the competing paradigms often do not compete on the same playing field according to the same rules and may require a re-valuation of scientific standards.

When paradigms enter, as they must, into a debate about paradigm choice, their role is necessarily circular ... each paradigm will be shown to satisfy more or less the criteria that it dictates for itself and to fall short of those dictated by its opponent [17].

But if new paradigms accept different base theories and methods of investigation and validate their data in different ways [18], then it is hard to see where the impetus for change arises. A fairly localized example of this process might be the switch in medical theory prompted by the discovery that Burkitt's Lymphoma as a neoplasia or cancer) was caused by infection with a certain virus stimulating immune cells to embark upon a course of disproportionate growth and reproduction. Prior to this discovery, infective diseases and neoplasia occupied different groups in our pathological classifications and new theories were developed to allow us to investigate the possibility that viral infection may play a causal role in cancer. Thus an answer previously marked wrong in any medical examination, viz. that some cancers are caused by infection, became right; what was untrue became true. This, however, is hardly a paradigm shift. At a basic level, the role of DNA both as part of the original genetic explanation of neoplasia and in viral action could be easily accommodated within medical theory.

Somewhat more radical is the challenge to the reductive basis of biomedicine posed by holistic therapy and the idea that the psychosomatic organism considered as a whole may influence even detailed molecular and patho-physiological processes contributing to disease in ways that cannot be defined by technologically driven medical laboratory science. If the effect of "holistic pseudo-science" becomes discernible enough to trouble us then we might begin to doubt the adequacy of the reductive biomedicine we now accept as unquestionable fact.

Similar points are made by Foucault in his comments on scientific revolutions:

... [their] extent and rapidity are only the sign of something else: a modification in the rules of formation of statements which are accepted as scientifically true. This is not a change of content (refutation of old errors, recovery of old truths), nor is it a change of theoretical form (renewal of paradigm, modification of systematic ensembles). It is a question of what governs statements, and the way in which they govern each other so as to constitute a set of propositions which are scientifically acceptable, and hence capable of being verified or falsified by scientific procedures. In short, there is a problem of the regime, the politics of the scientific statement [19].

Notice that Foucault is not speaking merely about competing descriptions or theories but rather "a regime of knowledge" governing the rules of formation of statements, rules that, although unstated, determine what counts as scientifically respectable according to legitimated concepts of observation, demonstration, confirmation of theory, validation of hypotheses, and so on. There is no straightforward assessment of theories here, "the regime" is much more inclusive and examining it poses searching questions about "the governance of statements" and the constraints on the corpus of knowledge that are sensitive to relations of power, both economic and political. These constraints control the discourses shaping our thoughts about human beings, their welfare, their reproductive activity, their "deviance", and their sanity. Therefore the socio-cultural and political aspects of medicine constitute a regime of truth informing clinical practice and the research "industry" central to contemporary biomedicine.

Foucault's analysis is sufficiently radical to call into question the very nature of biomedical science and its truth as distinct from dogma or opinion rooted in a culture that is based on financial interest, expediency, and professional power networks. Clinical science needs a philosophical response to this challenge robust enough to confront the ideologues of cultural relativism. There is a pragmatic response to this worry that recalls the Hippocratic view that medicine is a paradigmatic science with a starting point – human suffering, and a method – intervention, documentation and reflection, but there is an even more fundamental response based in philosophical semantics after Lacan. First the pragmatic response.

## A pragmatic response to relativism

Wittgenstein grounds meaning and truth in such a way that the meaning of any term is given by its use within some human practice [20] and truth results from the use of a term in accordance with the rules operating in that practice. Thus my statement to a trainee surgeon, "That is the carotid artery" when I have surgically exposed the carotid artery, is true and its contradiction false because I have used the term in accordance with the rules governing anatomical statements.

Wittgenstein likens words to tools and their meanings to the functions of tools, implying that the use of words from different discourses is like the use of tools from different activities. On this analogy, judgments about whether a set of words yielded knowledge would be like asking whether a hammer or a whisk did its job well. We might conclude that a hammer is a good tool in the work shed but not for cooking and vice versa for the whisk. Thus the aptness of a meaning to convey the truth about a situation is like the aptness of a tool for a task. Because our linguistically structured clinical activity has practical purposes, a pragmatic approach to the knowledge and truth is plausibly the one most suited to medicine [21]. But then the fact that different discourses have different purposes or activities associated undercuts the claim that a unitary standard of truth applies to all statements about health and illness [22].

The medical object is not given, it is constructed in the course of the activity of medicine, and in this sense it is a historically constructed object, the product or artefact of the relevant history of medicine. And so, too, is the practitioner or medical specialist, who comes to be that concrete individual by engaging in that practice.(1997, p63–4)

We should not, however, too easily slide into the idea that the functions identified and investigated by biomedical science are nothing but constructions of medical practices because a practice aims to make a difference in the world and medicine is an art addressed to the alleviation of human suffering.

Here we can find some guidance by examining a Hippocratic discipline – psycho-therapy – that has had to confront longstanding and thorny problems about its proper object. Lacan, remarking on the scientific status of psycho-analysis, notes that a basic minimum for any science is that it should have a definite object, although the object "changes, and in a very strange way, as a science develops" [23]. He notices the semantic ontology of physics where we have held on to something despite the fact that the *electron *of early modern physics is not that of contemporary quantum theory. But is there a path from that inquiry to our present topic: the nature of medical truth?

Disease-states are grounded in suffering so that the patient's suffering gives us a basic reference point for reflection on the medical object. However, such is the imperialism of scientific pathology and pathophysiology in medicine that many things of which patients complain, such as headaches arising from cervical spondylosis or chronic fatigue syndrome, are not recognized as diseases because we have no legitimated account of their pathogenesis and pathophysiology. Other things are regarded as diseases in that they represent deviations from biological norms (such as anemia, obesity, osteoporosis, or hamartomas) which are, as I have noted, to some extent artefacts of the diagnostic sciences and techniques of bio-measurement (for instance, obesity depends on identifying a person's Body Mass Index). Lacan examines briefly the ability to generate such formulae and notes that "a false science, just like a true science, may be expressed in formulae" [24] as is evident not only in alchemy, which Lacan mentions, but also other "false sciences" like phrenology, tarot, numerology, and palmistry. If, therefore, measurement, precision, and formulae do not distinguish true medical science from pseudo-science, we need other grounds for a robust concept of medical truth. To find them, more needs to be said about culture and meaning.

## Language, culture, and truth – the semantic circle

Contemporary continental philosophy follows structuralist conceptions of meaning in terms of the relationships of contrast and connectedness between signs. The central insight (closely relevant to understanding Lacan) is that language is based on a system of distinctions allowing us to group things according to our interests. Levi-Strauss argues that this is the most fundamental semantic move made by any language using group interweaving socio-cultural space with "natural" space [25] and it recalls Wittgenstein's claims that concepts are tied to our interests and meanings to the ways we use words [26]. It is evident that the differences to which a group responds and chooses to mark with signs are embedded in their ways of dealing with the world. Meanings, on this view, comprise differences between exploitable features of the environment which function as part of a system in which they have systematic structural relationships to one another. In fact this kind of pragmatic post-structuralism is a very plausible view; imagine for instance, an attempt to give an account of why it was important to learn the difference between a chisel and a screwdriver, or between parsley and paprika without mentioning the physical world and our bodily interactions with it.

We can therefore maintain that the contents of our thought are formulated in terms of the discourse which gives us a way of organising our responses to the environment in which that discourse has taken shape. Therefore the concepts and judgments made by a linguistic or cultural community in fixing the significance of various features of and occurrences in that environment frames their knowledge and the truths they are able to grasp. For instance, it would be hard to argue that the idea of romantic music could take serious hold in a society which did not have a history which spanned contrasting categories such as baroque, classical, or expressionist. Saussure remarks that the *signified *partakes of cognitive (and cultural) relations syntagmatically (or systematically) linked to *signifiers *such that structural distinctions mark the world in ways apt for structuring our interaction with it. Structure, on this view (as for Levi-Strauss), is unavoidably world-involving (Wittgenstein indicates this fact by using the term "grammar" to discuss the primitive language of the builder and labourer and its elaboration).

Therefore any plausible theory of meaning must pay attention to the way a subject categorizes the conditions in which she finds herself as is evident in the *old saw *of Fregean theory, "the morning star" and "the evening star" both of which refer to the planet Venus. A person may know what "the evening star' means without knowing this fact. Such a person knows the meaning of the term "the evening star" and that it is true that the evening star appears in the evening but not that the morning star and the planet Venus are also visible or that "the evening star' is not a star. All these terms – "morning", "evening", "star", and "planet" – mark things in the world according to our ways of thinking and not just according to the object being referred to by the terms involved. We can use them without realising that the object we are talking about or thinking in one way is also picked out by another way of thinking. These arguments add credibility to the strong thesis that culture determines language and through that productive relationship determines the truths available to a subject but the story should not end there.

Lacan offers us a way out of this circle of signification. He introduces a number of terms in order to articulate his theory about language and meaning.

The *tuche *is the actual lived encounter with the world, which Freud refers to as "the trauma" [27]. On this account the subject develops an understanding of what is around them in part by using signifiers made available by language to build or appropriate its structure into a representation of the world. For Lacan, the tuche – "the first encounter, the real, that lies behind the phantasy" – links us to what is around us.

*Language (Langue) *is a structured system forming the complex symbolic order "in which the world of things will come to be arranged" [28]. This structured system provides a human group with the categories used in thinking, categories predating any person's entry into the world. It is presided over by powerful figures who know how the world works, like the notional father who is identified with "the figure of the law" (whether an actual father or an icon) and represents the authority of the socio-cultural nexus which determines the significations that should be applied to things [29]. Lacan (following Levi-Strauss) notes that the symbols form a network underpinning the exchanges of a human group [30] and for any given human being this network transcends the individual to form the context shaping the individual psyche: "human psychology cannot be conceived in the absence of the function of the subject defined as the effect of the signifier" [31]. The thoughts and projects of human beings are framed in terms of this shared system.

*Speech (Parole) *is the medium of interpersonal exchange derived from language and in it I find myself mirrored by others and their descriptions of me. I have an important (and creative) role in this discursive project of speaking life into language because in use [32], including *my *use, words take on life or meaning.

*The signifier and the signified *therefore are intrinsic to the idea of truth as a relation between the subject and the world in which the subject lives. But notice that the semantic content associated with a given sign may evolve and change with changes in the language in which that sign has life. What is more, the discursive history of the subject, which is the shaping ground of the psyche, is part of an evolving project (by a human group) in which truth must be brought into being, both truth about the world and truth about themselves. Lacan, by accepting a structuralist view of meaning and also emphasising the *tuche *or encounter with the real, puts an interesting twist on the Freudian "thing", which Freud himself directly borrowed from the Kantian "*ding an sich*". The structure of language delimits the encountered "thing"(moment, trauma, tuche) as thinkable but the thing in itself affects me apart from its thinkability.

This insight shows us where we might go beyond the circle of culture, signification and socially constructed truth. The tuche or encounter with the real which is close to Freud's *trauma *or disturbing *event in itself *is signified by the subject but no-thing ever can be fully captured by signification as there is always that aspect to an encounter which has a causal effect on one but does not find a home in one's system of signification. (I would explicitly link the inexpressibility of that elusive relation between the subject and the object to the causal properties of the encounter.) We can, in fact, generalize this observation and notice that there are aspects to any event which are part of the totality of the world as it appears in, but is not fully captured by, the totality of my current significations deployed in the situation concerned and their symbolic connections.

We might repeat this generalizing move and notice that the encounter with the real is also an encounter with the object as it is encountered by and signified by others whose subjectivity *vis-à-vis *the object is necessarily incompletely accessible to any given subject, through conversation and what is revealed there. That fact potentially leads one to feel that there is more to an encounter than one can currently (or indeed ever fully) comprehend and the resulting psychic incompleteness is potentially disturbing or worrying. Lacan argues that the attempt to get control of this elusive aspect of the encounter with the real is the basis of repetition whereby something that outstrips the ability of my signifiers to capture it causes psychic fascination or "sticking".

*Repetition*, (regarded by Lacan as one of the four basic concepts of psychoanalysis), is the tendency to be drawn back to an event or focus of mental disquiet that has not been satisfactorily integrated into conscious thought. It indicates a gap between the encounter and the signification: "repeating ... is related to ... remembering" but is not encompassed in narrative memories because the real adds an elusive quality to the repeated encounter "the real is that which always comes back to the same place – to the place where the subject in so far as he thinks, where the *res cogitans*, does not meet it" [33]. The Cartesian allusion is telling in that Lacan undermines the twin theses that the contents of the mind are transparent to conscious reflection and that those contents are solely in terms of existing significations. Lacan clams that they are not transparent and their content goes beyond what signifiers can (as it were) "swallow" or capture in intentional terms.

The next move picks up the point that repetition shows itself, par excellence, in a restless driven-ness to close the gap between encounter and signification. This gap means that whereas praxis adds an element of practical involvement in the real world to our significations of things, the tuche adds a similar subjective involvement.

## Truth and the encounter with reality after Lacan

Any praxis occurs within and defines a field of activity in which its practitioners work and its concepts enable one to locate (cognitively), interact with, and exploit the objects constituting that field. But rather than finding that legitimation and power define experience (as in strong forms of socio-cultural constructionism) a further possibility is found in the encounter with the real that escapes signification and provokes repetition. The repetition is particularly likely if the practitioners are attuned to suffering and the tools provided by an epistemic regime do not adequately meet the actual sufferings of human beings (as in Hippocratic times).

Notice the structure of the argument:

1. Truth is a product of language and the encounter with the real.

2. Language is a product of culture.

3. The encounter with the real may demand more than language can provide.

4. It particularly does so where our praxis is motivated towards a certain kind of adequacy.

5. A Hippocratic praxis is motivated to provide an adequate response to suffering.

6. Theoretically doctrinaire medicine may not be such a response.

7. Hippocratic practice is driven to go beyond its tools of signification and find new truths to be engaged with and given articulate form.

We can add Foucault's acknowledgement that not all sciences are on an equal footing in their vulnerability to "social forces". He argues that sciences such as theoretical physics and organic chemistry are relatively immune to such influences. But what makes a science more likely to restlessly search for truth and less likely to be subject to power relations shaping its discourses of validation?

Medicine provides an interesting case study. On the one hand it is practical and has palpable results so that the cycle of conceptualization, intervention, monitoring of results, and refinement of theory can be kept in touch with real world events at the coalface that is the clinic. Toulmin (invoking Wittgenstein's concept "form of life") speaks of a medical *Lebenswelt *(life-world) comprising *Lebensformen *(forms of life) or "different substantive enterprises that have survived the pragmatic tests to which they were subjected in the evolution of those enterprises" [34]. Therefore the Hippocratic foundations of medicine provide a reality check on medical truth.

On the other hand, there is a discursive regime conditioning contemporary biomedical science the outlines of which I have already sketched. This regime exclusively fosters a relatively mechanistic conception of human function. It has invisible but palpable control of the medical research industry and over many facets of clinical life, including the legal constraints on best practice – which tend to track technology rather than clear evidence of efficacy. Given the interests converging at the point where medicine meets power, the political process, and economics, we might worry that medicine itself is more prone than other branches of science to ideological and axiomatic distortion. Even the focus on evidence might be a convenient and effective means by which orthodoxy cements its hold on the profession and thereby on the human population it serves.

If a naive, or modestly critical, realist view of scientific knowledge is unsustainable, wherever the regime of truth is shown to have significant political, economic, and cultural determinants, how can we justify the view that, in medicine, we are seeking a knowledge transcendent truth (of the type beloved by realists of all stripes)?

## Orthodoxy, trauma, and repetition

Foucault's challenge to the conventional notion of truth and his focus on power in determining what is accepted as valid knowledge are disturbing to those wanting to escape cultural idealism. Can we, in the face of such doubts, erect any boundary (even if it is fuzzy, shifting, and difficult to define) between those aspects of medical knowledge which are vulnerable to post-Kuhnian and Foucauldian critiques and those which are not?

We can find some aspects of what we need (as Pellegrino claims) in the Aristotelian idea of a *techne *– an art informed by technical knowledge and reflection showing a dynamic interplay between praxis and conceptualization [35]. This approach (as Toulmin showed) has clear conceptual links to Wittgenstein's analysis of meaning and truth arising within language games and forms of life and evades, to some extent, the challenge directed at traditional epistemology.

Traditional epistemology posits a relation between two independent terms – the world and the thinker mediated by the ideas of the thinker. Thus the traditional epistemic problem: if the thinker only knows about the world through (theoretically informed) ideas (and observations), how does she know what things are really like objectively? Traditional idealists and many post-modern writers conclude from this problem that ideas, language, or discourses are internal and self-regulating such that different discourses result in hermetically sealed constructions of reality. Truth then becomes located within a particular discourse so that there can be apparently contradictory claims arising within different discourses. A *lebenswelt *and its *lebensformen *are, however, different particularly when focused on something as complex and multifaceted as human suffering. Reality constantly engages the healer so that medicine could be thought of as a kind of institutional neurosis in which the intractability of suffering demands repetitive attention to our inadequacies in dealing with it. Any less ethically driven response then becomes impure or immoral and compromises the truth of our proceedings (for Lacan, a feature of true science is the "purity of soul of the operator" [36]).

The pragmatists direct our attention toward the purposes and interests served by knowledge and the cultural critique of scientific realism bites at just this point. But this concession, as in general epistemology, need not undermine a substantial conception of truth (as related to purpose driven cognitive maps of a domain of praxis) particularly when, after Lacan, we examine the tuche.

Truth is a function of a given cognitive map (and therefore *Lebenswelt*). Because it is advantageous not to have a series of isolated and impressionistic maps of any domain of activity, human thinkers tend to make connections between intersecting (and sometimes irreconcilable) concepts and constructions. Each true thought fits well into some cognitive map of the situation and, to some extent validates the use of that map at that time. One hopes that some at least of the true thoughts one has in such a clinical situation allow us to move with assurance and propriety in our actions toward the patient. On that basis, a thought – for instance about the meaning of an illness event in the whole context of a person's life among others might be a good thought with truth-bearing and adequately action-guiding qualities. It has, we could say, both epistemic and practical value which, for a pragmatist, largely coincide.

I have, following Lacan, claimed that a subject with the appropriate attitude towards medical truth is troubled by human suffering especially if medical science finds it neither understood or even signifiable in a way that allows us to work towards an answer. This ethical sensitivity potentiates a tuche – a residue that may escape scientific signification – and a properly attuned soul will return again and again to the "wound in his clinical psyche" that is his or her inability to respond to the suffering.

Therefore moral virtue and epistemic virtue are intertwined. It takes a certain kind of person (one with what Nussbaum calls a developed "sense of life") to achieve the relevant openness to experience [37], and it takes not only a certain kind of cognitive skill to conceptualize what experience reveals but also an ability to deconstruct and escape the validated links that are created and legitimated by an area of discourse. Clinical praxis involves subjective bodies and their intersecting trajectories, a reality that engages a clinician with patients, so that the suffering of the patient spurs them to try out ways of conceptualizing what is going on until they discover regularities that are worth pursuing. The result is Hippocratic knowledge, that multifaceted set of skills that can respond adequately to suffering despite a very imperfect framework of theory at any given point in that enterprise.

## Truth, virtue, and discovery

These vague moves towards a post-Kuhnian and post-structuralist epistemology for medical science take us back towards the epistemic and practical values endorsed by Hippocrates. I have used the term "epistemic virtue" and linked epistemic and moral virtue, in a way that Plato and Aristotle both could be taken to suggest, as different facets of the same jewel. But is this position robust in the face of a post-modern critique of clinical practice and its scientific products?

Foucault argues that power and relations of power control the legitimation of statements within any given discourse. This applies a fortiori in medical discourse where a set of power relations confer an exalted status on the conceptualizations and validated judgments of doctors as distinct from others. Doctors decide "what is really going on", "the good of the patient," "good evidence for this or that", "a reasonable therapeutic option", and so on. The story of the patient may be left out of this set of discursive constructions and, even more radically, the privileged discourse excludes the patient's opinion whether the medical story actually fits his illness. If post-modernism teaches us any lessons they would include the idea that the thoughts or discursive constructions of situations that most deserve our endorsement are those that transcend and reveal power imbalances. This implies a moral constraint whereby true knowledge extends a discourse and liberates it from the regime put in place by a power group (such as the biomedical industry). Liberation, in the case of clinical knowledge should enable it to be owned equally by the sufferer and the healer.

It emerges that the proper evaluative aspects of clinical practice, the moral commitments of good doctors, and the sharing of information and power, are inseparable from the truths of the clinic. Lacan's remark about the purity of the soul of the operator is therefore vindicated (for medicine at least) in that clinical practice is best when the relation between *the good *and knowledge is clearly appreciated (but not quite as Plato envisaged it) in ways that are not always apparent in other areas of scientific endeavour.

Hippocrates thought that medicine is a great exemplar of science in general because it methodically and sensitively charts the accumulated wisdom that comes from engaging with the realities of the human condition. The inseparability of praxis from science that is underscored by that fact implies that real knowledge is more to do with acting well than with building theoretical structures dominated by an elite controlling a regime of truth. Truth cannot be separated from the encounter with the real and the care for human suffering with which the Hippocratic writers attempted to infuse it. Lacan reinforces this crucial point just where fuzzy relativism is often thought to loom large.

The intrusion of post-modern thinking into medical ethics introduces the thought "that illness is no longer a purely biological state – no longer a brute fact of nature – but rather something in part created or interpenetrated by culture" [38]. The idea that disease is a "biocultural" phenomenon (Morris' term) suggests that medical truth goes beyond the structure of signs, texts, and political functions that control biomedical scientific truth and seeks the story that has been appropriated by that framework. But then it goes beyond the medical story to look at the encounter with reality that occurs in a human life and inscribed itself on the body of the individual concerned. This body has fallen under the medical gaze and is not doing that which it normally does and we need to appreciate the alienation that has crept into the subjective body to understand the patient's dis-ease.

An openness to individual meaning and beyond that to the dynamic encounter between the patient, the doctor, and the real might, therefore, be expected to reveal a truth that goes beyond medicine.

There is, therefore a significant convergence between, on the one hand, the epistemic and moral virtues of the Hippocratic tradition (marked by compassion, empathy, and pragmatism) and, on the other, the conception of clinical science that emerges from the post-modern critique. This convergence allows us to move ahead in specifying some of the hallmarks of virtuous practice for the Hippocratic professional in a post-modern era.

## Conclusion: medicine and truth

Orthodox medicine has followed the Hippocratic model gaining knowledge from cumulative experience of the cases arising in clinical practice. However the intrinsic power of academic and institutional medicine has made it theory bound and paradigm dominated reinforcing its tendency to occupy the epistemic high ground and to appeal to demonstrated efficacy within the healing ethos.

Medicine has adopted the scientific model of truth but that model is challenged when the tuche declares itself and alerts us to the fact that our categories are not the thing in itself but only a provisional and partial signification of it. Some stories serve to upset that paradigm in thought-provoking ways.

### The powerful placebo

The patient had a long-standing and refractory clinical depression and had tried most of the available anti-depressants without effect until she was enrolled in a drug trial. The new treatment gave her dramatic and sustained improvement, much to the relief of her clinical caregivers. However she had, in fact, been in the placebo arm of the trial. As ethicist, I was asked what her treating clinicians should tell her.

### The postponed craniotomy

I was about to do a craniotomy for a cerebral aneurysm which had caused a sub-arachnoid haemorrhage. It was Sunday afternoon and as I was about to scrub up and start, the anaesthetist mentioned that the patient had responded strangely during anaesthetic induction (as he had throughout the 48 hours since his haemorrhage.) The images of my discussions with him and his appearance and responses passed before my mind, I felt that he would suffer severe cerebral vasospasm and a stroke if I proceeded and that I should delay the operation. I said to the O.R. team that I did not feel right about doing the operation despite all their preparations. The senior operating theatre nurse said "If you don't feel right about it, you should not go ahead." I thought to myself that I was a good evidence based practitioner and that this kind of thing was not part of rational clinical conduct but I bowed to my misgivings and cancelled the operation. 24 hours later, the patient was a different man and had his surgery without complications (which, of course, might have happened anyway).

In each of these cases we enter a domain in which the patient as a subjective body encounters the real and is touched by it. We find that we are drawn beyond the comfort of the framework of theory and observation created by scientific biomedical culture and realize that there are more things in heaven and earth than are conceptualized in our philosophy. We are then led to try and understand what it is that our openness to life situations has brought us into contact with. The recognition that we are "in touch" with something real and a creative pursuit of what eludes us then become a path to truth in the face of the trauma that is human suffering.

## References

[B1] Lloyd G (1978). Hippocratic Writings.

[B2] Lacan J, Sheridan A (1981). The four fundamental concepts of Psycho-analysis.

[B3] Kuhn TS (1962). The structure of scientific revolutions.

[B4] Feyerabend P (1975). Against Method.

[B5] Newton-Smith W (1981). The rationality of science.

[B6] Hacking I, Galison P, Stump D (1996). The disunities of the sciences. The disunity of science.

[B7] Reilly D (2001). Enhancing human healing. British Medical Journal.

[B8] Putnam H (1973). Meaning and reference. Journal of Philosophy.

[B9] Kripke S (1980). Naming and necessity.

[B10] Hudson R (2001). Discoveries, when and by whom?. British Journal of the Philosophy of science.

[B11] Boorse C (1975). On the distinction between disease and illness. Philosophy and Public Affairs.

[B12] Megone C (1998). Aristotle's function argument and the concept of mental illness. Philosophy Psychiatry and Psychology.

[B13] Gillett G (1999). The Mind and its discontents.

[B14] Fulford W (1989). Moral theory and medical practice.

[B15] Foucault M, Rabinow P (1984). The Foucault reader.

[B16] Kristeva J (1995). The new maladies of the soul.

[B17] Kuhn TS (1962). The structure of scientific revolutions.

[B18] Kuhn TS (1962). The structure of scientific revolutions.

[B19] Kuhn TS (1962). The structure of scientific revolutions.

[B20] Foucault M, Rabinow P (1984). The Foucault reader.

[B21] Wittgenstein L, Anscombe G (1953). Philosophical Investigations.

[B22] Toulmin S, Carson R, Burns C (1997). The primacy of practice: medicine and post-modernism. Philosophy of medicine and bioethics.

[B23] Davidson A, Galison G, Stump D (1996). Styles of reasoning, conceptual history, and the emergence of psychiatry. The disunity of science.

[B24] Lacan J, Sheridan A (1981). The four fundamental concepts of Psycho-analysis.

[B25] Lacan J, Sheridan A (1981). The four fundamental concepts of Psycho-analysis.

[B26] Levi-Strauss C, J, Weightman D (1966). The savage mind.

[B27] Wittgenstein L, Anscombe G (1953). Philosophical Investigations.

[B28] Lacan J, Sheridan A (1981). The four fundamental concepts of Psycho-analysis.

[B29] Lacan J (1977). Ecrits.

[B30] Lacan J, Sheridan A (1981). The four fundamental concepts of Psycho-analysis.

[B31] Levi-Strauss C, Paul SO, Paul R (1967). The scope of anthropology.

[B32] Lacan J, Sheridan A (1981). The four fundamental concepts of Psycho-analysis.

[B33] Wittgenstein L, Anscombe G (1953). Philosophical Investigations.

[B34] Carson R, Burns C (1997). Philosophy of medicine and bioethics.

[B35] Toulmin S, Carson R, Burns C (1997). The primacy of practice: medicine and post-modernism. Philosophy of medicine and bioethics.

[B36] Pellegrino E, Carson R, Burns C (1997). Praxis as a keystone for the philosophy and professional ethics of medicine. Philosophy of medicine and bioethics.

[B37] Carson R, Burns C (1997). Philosophy of medicine and bioethics.

[B38] Nussbaum M (1990). Love's Knowledge.

[B39] Morris D (1998). Illness and Culture.

